# Chatbot Outreach in Value-Based Preventive Care: Retrospective Analysis

**DOI:** 10.2196/81370

**Published:** 2026-02-12

**Authors:** Jincy Chacko, Yuane Jia, Kimon Stathakos, Kathleen Mazza, Doran Kim, Michael Diefenbach, Lorena Carlo

**Affiliations:** 1 Northwell Health New Hyde Park, NY United States; 2 Department of Health Informatics School of Health Professions Rutgers University Piscataway, NJ United States; 3 Department of Interdisciplinary Studies School of Health Professions Rutgers University Newark, NJ United States

**Keywords:** chatbot outreach, digital health, health communications, health equity, preventive care gaps, value-based care

## Abstract

**Background:**

As health care delivery shifts toward value-based care, proactive strategies to close preventive care gaps are essential. However, patient engagement remains suboptimal due to logistical, behavioral, and socioeconomic barriers. Traditional outreach methods, such as phone calls, emails, and postal mail, have long been used, but emerging digital approaches, such as chatbot-based messaging, offer potential advantages in scalability and personalization. Their comparative effectiveness, however, remains underexplored.

**Objective:**

This study aimed to evaluate the effectiveness of chatbot outreach compared with traditional communication methods (phone, email, mail, and multichannel) in promoting compliance with preventive screenings and wellness visits defined by the Healthcare Effectiveness Data and Information Set and the Centers for Medicare & Medicaid Services guidelines.

**Methods:**

This retrospective study evaluated patient outreach campaigns conducted from 2021 to 2023 across an integrated health system in New York. The final analytic sample included 50,145 care gaps from 41,959 eligible participants, predominantly female (29,989/50,145, 60%), White (31,857/50,145, 64%), with mean age ranging from 49.36 to 72.81 years over the study period. All participants were residents of New York state, and 81% (40,553/50,145) maintained an active relationship with a primary care provider during the participation year. Outreach modalities included automated chatbot SMS text messages, nonautomated phone calls, and organization-led email or mail campaigns. Participant data were enriched with social vulnerability scores to account for community-level disadvantages. Exposure was defined as the outreach method (chatbot, phone, email, mail, or multichannel), with assignment based on engagement history and operational protocols. The primary outcome was care-gap closure or compliance with identified measure gaps annually. Logistic regression and chi-square analyses examined associations between outreach method, patient demographics, primary care physician relationship, social vulnerability index (SVI), and compliance.

**Results:**

Phone outreach consistently achieved higher compliance than chatbot or multichannel outreach across most groups and years. Chatbot messages outperformed phone calls only in diabetes care in 2023 (odds ratio [OR] 1.81, 95% CI 1.48-2.21; *P*<.001). Primary care physician continuity remained a strong predictor of gap closure, especially in primary care (ORs ranged 1.36-2.61; *P*<.001). Higher SVI quartiles were associated with lower compliance in blood pressure, cancer care, and diabetes care groups; however, primary care outcomes showed higher odds of compliance in the third quartile of SVI, contradicting the typical linear-deprivation narrative. Women, Hispanic or Latino individuals, and Asian patients demonstrated higher odds of compliance in some groups and years.

**Conclusions:**

Outreach modality is an important, modifiable factor in preventive care adherence. While phone-based outreach remains the most effective overall approach, chatbot-based strategies may have targeted applications in digitally engaged populations such as the diabetic group. Segmented, equity-informed outreach strategies that integrate technology, patient preferences, and primary care continuity are essential to achieving high-impact, scalable outcomes in value-based care settings.

## Introduction

### Background and Rationale

The development of health care delivery in the United States over the past decade has increasingly centered on value-based care (VBC), which emphasizes quality, outcomes, and cost-effectiveness over service volume. Promoted by the Centers for Medicare & Medicaid Services (CMS), the value-based preventive care concept represents a focused application of the VBC framework that prioritizes proactive prevention and early disease detection, aligning financial incentives with population health goals [1,2]. This approach is particularly relevant for managing chronic diseases and addressing preventive care needs in large, diverse patient populations [3].

Despite widespread consensus on the benefits of preventive services such as annual wellness visits, cancer screenings (CXs), diabetes care (DB), and blood pressure (BP) monitoring, patient adherence remains suboptimal [4-6]. Barriers to engagement include socioeconomic challenges, low health literacy, lack of awareness, and fragmented communication between care providers [7,8]. Health systems have historically used outreach methods such as telephone calls, emails, and postal mail to notify and engage patients with gaps in preventive care. However, these approaches can be resource-intensive and vary in their reach and effectiveness, particularly among hard-to-engage populations [9].

Emerging digital outreach tools, including automated chatbots and SMS text messaging, may offer a promising alternative for improving engagement at scale. These technologies can support bidirectional communication, personalize patient interactions, and reduce administrative burdens [10]. Several public health campaigns, particularly during the COVID-19 pandemic, have demonstrated the feasibility of chatbot-based outreach in promoting vaccine uptake and disseminating timely health information. However, the use of these tools for broader preventive care engagement, particularly in chronic disease management and CX, remains underexplored [11].

Based on a previous systematic review conducted on this topic by Chacko et al [11], the current state indicates a limited body of evidence on chatbot outreach beyond immunization programs, highlighting a need for further research into their effectiveness, cost-efficiency, and equity impact. Studies reporting on outreach mechanisms for preventive care have varied in their implementation approaches and study methodologies. Therefore, drawing a generalizable conclusion is not possible. It is uncertain if benefits of chatbot outreach apply uniformly across various demographic and clinical groups. Moreover, the integration of these tools into large health systems workflows may present unique challenges that remain underexplored in the literature [11].

### Objectives

To address these gaps, this study aims to evaluate the impact of emerging technology, such as chatbot-based outreach, on preventive screening uptake compared to conventional methods, such as outreach via phone calls and email communications. The authors posited that the use of chatbots in preventive care will significantly increase the uptake of screening for annual wellness visits, hypertension, diabetes, and CXs compared to traditional methods of communication. Conducted at a large integrated delivery network in New York, the study focused on patients identified as having care gaps using the Healthcare Effectiveness Data and Information Set (HEDIS) and CMS preventive screening criteria. This study builds upon recent findings that suggest automated outreach tools may offer scalable and equitable solutions for preventive health engagement [11]. By evaluating their effectiveness across multiple screening domains, which included CXs, DB, and hypertension screening, along with primary care (PC) measures such as annual wellness visits, the study contributes to the growing body of evidence informing digital outreach strategies in VBC-aligned care delivery. Findings from this evaluation may help guide future investments in digital health infrastructure and support the implementation of targeted, technology-enabled outreach programs.

## Methods

### Study Design

This study retrospectively evaluated data collected from VBC outreach activities conducted over a 3-year period, from 2021 to 2023. Members identified by their insurance carrier as having care gaps, attributed to the health system, were targeted for outreach. Outreach was conducted using phone calls, web-based chatbots, email messages, mailed letters, and sometimes a combination of these methods. The strategy for outreach method allocation was dependent on operational capacity and technology adoption. During year 1 (2021), due to reduced operational capacity following the 2020 pandemic year, new technology was piloted to aid in outreach. The subsequent years’ attribution strategy was adjusted to maintain a balance between outreach methods with increased volume of gaps, capacity in the workforce, and efficiencies found in technology adoption. Multichannel outreach involves the sequential use of 2 or more methods within the same measurement year when initial outreach attempts fail to result in engagement. Due to the inability to attribute the outcome to any single method alone, outreach using multiple methods was grouped into 1 category. Outreach method assignments followed operational protocols and capacities that varied each year, rather than being randomized. Therefore, this study is exploratory in nature, focused on understanding trends that may drive empirical decisions about the modality of outreach in the future.

### Data Sources

Participant demographic information was collected from the health system’s electronic medical record (EMR). Outreach process measures and final compliance data were collected 3 months post each study year from the respective operational teams performing outreach. These data included an indicator for participant relationship with primary care provider (PCP) within the health system and the total number of care gaps for the given year per participant. Deidentification was completed before the data were shared with researchers. The social vulnerability index (SVI) was acquired from the 2022 dataset compiled by the Centers for Disease Control and Prevention [12]. The census tract level SVI was mapped to participant zip code with the aid of cross-mapping from the Department of Housing and Urban Development, accessed in June 2024 [13].

### Inclusion Criteria

Participant eligibility for the identified gap measures was a primary inclusion criterion. Clinical criteria for eligibility are industry-defined guidelines (electronic Clinical Quality Measures), maintained by CMS [14], or HEDIS specifications provided by the National Committee for Quality Assurance [15], with changes published each year publicly. These define the required age ranges, diagnosis inclusion, and completion intervals for preventive services such as wellness visits, CX, BP, and DB. The other important criterion determining inclusion in the final analysis was having at least one outreach attempt using a phone, chatbot, email, or letter during the measurement year. As the study focused on understanding the efficiency of outreach methods, the population that received no outreach was outside the scope of this study.

### Exclusion Criteria

Preventive measure groups and individual measures with an insufficient sample size (n<10) were excluded due to the need for valid statistical and comparative testing. Records with unknown outcomes (ie, unverified or missing documentation of care gap closure) or missing demographic variables (age, gender, race, ethnicity, and address [needed for SVI grouping]) were excluded. For example, if there was no known address for the patient documented, it would not be possible to map an SVI and include the participant in the stratified analysis, and therefore, such participants’ data were removed from all study datasets before any analysis was conducted. Process measures such as documented patient engagement steps (eg, message opened or call answered) were collected and used to define successful outreach delivery. Unsuccessful outreach deliveries (eg, in case of erroneous contact information) were excluded.

The dataset remained representative of the population served by the health system and met eligibility criteria for each measure throughout this process. Four measure groups remained eligible for analysis after the applied exclusions: PC, BP, CX, and DB. Multimedia Appendix 1 provides the distribution of participants and measure gaps within each group.

### Data Management

**Figure 1:**
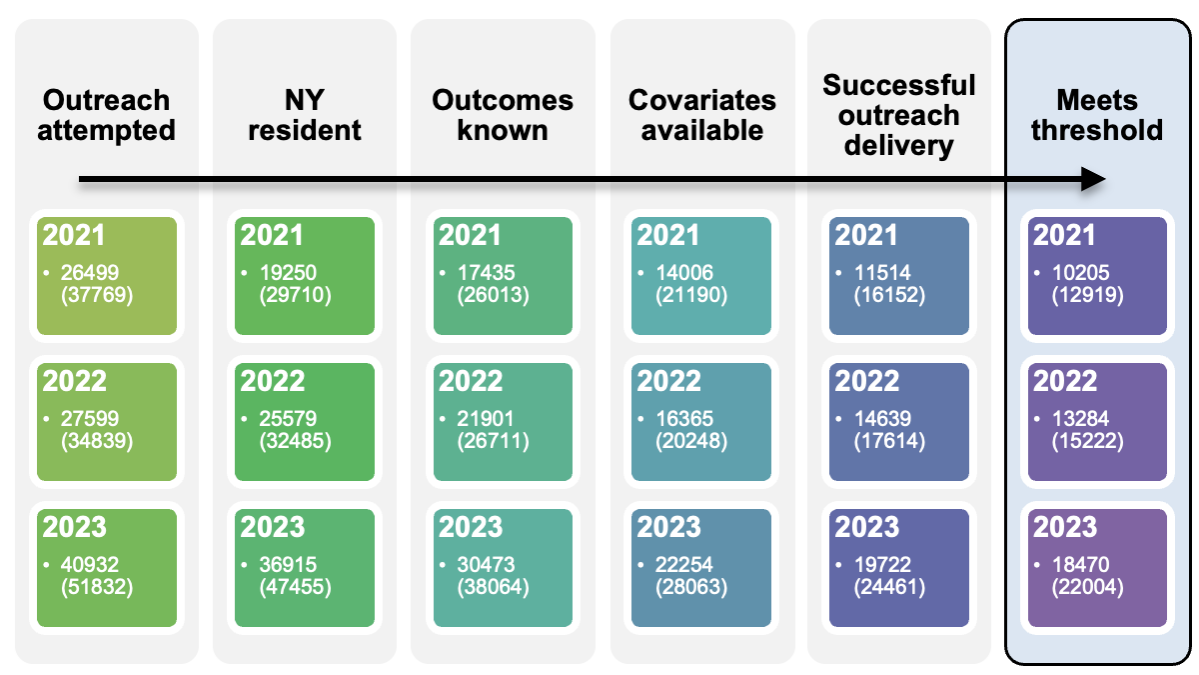
Participant and measure gap flow diagram for each year in the study, listing participant volume first and gaps (n) in parentheses. NY: New York.

Deidentified data were aggregated at the patient-gap-year granularity using Microsoft Power BI [16]. Individual preventive measures were grouped into care domains (eg, PC, CX, etc) based on clinical relevance and alignment with HEDIS and CMS quality measurement categories. The grouping was determined by the study team using established measure definitions to facilitate domain-level comparisons. The case of multiple SVIs mapped to a single participant due to geographic overlap in the data was resolved by assigning the SVI from the most populous county to the participant, which is a common approach in health care research. Figure 1 shows the flow of participants and gaps volume through the applied exclusions.

### Study Variables

The primary independent variable, also referred to as the outreach method or intervention, was coded as 1 of the 4 single-channel methods (phone, chatbot, email, and mail) or multichannel to indicate any combination of these methods. Multichannel methods were grouped because the sample sizes in individual groups did not meet the statistical validity requirements for comparison.

The primary outcome or dependent variable evaluated was the completion of or compliance with the identified measure, measured 3 months following each study year (eg, March 2022 for the study year of 2021). It was documented separately for each year of the study, 3 months post end of year, and represented as a binary variable, indicated by 1 for “yes” and 0 for “no.” The primary outcome was collected as part of the operational process of verifying completion of or compliance with the measure gap within the reporting period. This may include a verification by patient report, EMR validation, or a confirmation by payor data sharing. The authors collected this information retrospectively from data reported to governing agencies for each year.

Demographic data, including age, gender, race, ethnicity, and the SVI, were used to study covariates that could potentially impact the outcomes of outreach. Additionally, covariates included the presence of an active relationship with a PCP (defined as having a designated PCP of record within the health system and at least 1 documented encounter with that provider or associated practice within the preceding 18 months) and the burden of unmet care (defined as the total count of gaps for each participant per year).

Among the continuous variables, age was capped at 89 years to prevent reidentification. The count of gaps variable was used to assess potential imbalances in record weighting within the grouped measure files, as the datasets were analyzed at a gap level (n=1 gap per participant) rather than at a participant level. Tables S1-S4 in Multimedia Appendix 2 demonstrate the range and mean of the count of gaps per year for each measure group.

Categorical variables included demographic factors, gender, race group, ethnicity, socioeconomic factors, SVI, and active PCP relationship. Gender was coded into male/female as collected in the EMR. Race was grouped into White, Black or African American, Asian, and other. The other race group included Pacific Islander, Alaskan, and Native Americans, as well as patient self-reported “other” race identity. Ethnicity was only collected as a bivariate containing options for Hispanic and Latino or not Hispanic or Latino. Each participant was assigned an SVI quartile (1-4), where quartile 1 was the group with the least social vulnerability, and quartile 4 was the group with the most social vulnerability.

### Statistical Analysis

All statistical analyses were conducted using IBM SPSS Statistics for Windows (version 29.0) [17]. The dataset was divided into measure-group files for each year. Analysis was performed for each measure group, with BP as an exception because it contained only 1 measure. Textbox 1 provides a list of subgroups (measures) within each measure group that were included in the final analysis. Descriptive statistics and contingency tables verified adequate cell counts (n≥10) for bivariate analyses per measure group, and any variables not meeting the threshold were excluded from final analysis for the respective measure group [18,19]. Notably, SVI quartile 2 often did not meet the valid n threshold, as was the case when the outreach method was email only. These were excluded from statistical analysis. This conservative approach ensured the robustness of the reported associations while maintaining data integrity across all measure groups. Effect sizes are reported as odds ratios (ORs) with 95% CI and *P* values. Categorical variables were converted into dummy variables using reference coding (eg, phone outreach, male, White, non-Hispanic/Latino, SVI quartile 1, and no active PCP as baseline categories) before logistic regression analysis.

**Measure groups and subgroups 2021-2023.**

**Primary care**

- Annual wellness visit
- Pediatric well-care visit

**Blood pressure (single measure only)**

**Cancer screening**

- Breast cancer screening
- Cervical cancer screening
- Colorectal cancer screening

**Diabetes care**

- Hemoglobin A1c control <8.0% in diabetes
- Hemoglobin A1c poor control >9.0% in diabetes
- Hemoglobin A1c testing in diabetes
- Nephropathy assessment
- Retinal eye examination
- Kidney health evaluation for participants with diabetes

Collinearity was assessed between included variables, which led to the exclusion of the insurance group from the final analysis due to its high correlation with age in this dataset. Chi-square tests identified preliminary predictors among the categorical variables, after which a 2-step backward stepwise logistic regression was performed. Step 1 entered all demographic variables and PCP relationship. Step 2 introduced outreach modality to quantify its independent association with the final compliance outcome, that is, gap closure after controlling significant covariates in the previous step. A chi-square test was also conducted between the PCP relationship variable and outreach methods to examine any existing associations before both were included in the regression model. Statistical significance was set at α=.05.

### Ethical Considerations

The Northwell Institutional Review Board reviewed this study protocol and approved it on August 15, 2024 (HSRD24-0151), with a waiver of consent requirements in accordance with research involving deidentified secondary data. No patient contact or intervention occurred as part of the study, and all data were fully anonymized prior to analysis. To minimize analytic bias, eligibility and exclusion criteria were applied consistently using standardized definitions from HEDIS and CMS electronic Clinical Quality Measures.

## Results

### Participant Characteristics and Descriptive Statistics

After applying exclusions, the analytic dataset contained 12,919, 15,222, and 22,004 care gaps for 2021, 2022, and 2023, respectively, bringing the overall volume of gaps to 50,145. Gap volumes increased by approximately 18% (15,222/12,919) in 2022 and 45% (22,004/15,222) in 2023, with PC accounting for 47% (23,481/50,145) of gaps, CX 31% (15,335/50,145), BP 12% (5891/50,145), and DB 11% (5438/50,145). A total of 81% (40,553/50,145) of gaps came from participants who had an active relationship with the PCP during the participating year. Women predominated in all groups except diabetes in 2021-2022, and the cancer cohort showed an expected skew due to female-targeted measures. Among the 50,145 gaps, participants mostly belonged to the White racial group at 64% (31,857), followed by other 18% (8812), Black at 12% (6230), Asian at 6% (3246), and Hispanic/Latino at 16% (8199). The mean age for the PC group declined from 72.8 (SD 11.6) years in 2021 to 60.2 (SD 27.1) years and 49.4 (SD 30.5) years in 2022 and 2023, respectively, resulting from the gradual inclusion of the pediatric subgroup. All other measure groups had a mean age range from 56.4 to 66.3 years. The average gaps per participant were below 2 for all measures except DB, which had 2.9 gaps per participant in 2021, 2 in 2022, and 2.2 in 2023. See Multimedia Appendix 2 (Tables S1-S4) for detailed distribution for all 3 years.

Outreach included phone, chat, email, and multichannel approaches. Email was only valid for PC in 2021 and DB in 2023. Multichannel cohort met criteria in PC and CX across all years and DB and BP in 2023. Outreach shifted from phone-dominated in 2021 to greater chatbot and multichannel use by 2023. Multimedia Appendix 2 displays this trend with detailed values for each group.

### Compliance Trends and Bivariate Associations

Multimedia Appendix 3 indicates the compliance rate for all 3 years across the measure groups. All measure groups indicate the highest compliance rate in 2021 and a subsequent decrease in 2022. While other measure groups have increased compliance in 2023, possibly indicating a postpandemic stabilization, compliance in the BP group continues to decrease this year. This decrease, however, is relative, as BP continues to be the group with the highest compliance rate each year (1042/1486, 70% to 1440/2550, 56%), while CX is the group with the lowest compliance rate (1698/4264, 40% to 1600/7656, 21%).

Across all measure groups, the outreach method showed a significant association with compliance. The PC group demonstrated the strongest relationship in 2021 with a large effect size (*χ*^2^_3_=1767.8; *P*<.001; V=0.57). Though this association persisted for all 3 years, its magnitude decreased, showing a moderate effect size in 2022 (*χ*^2^₂=220.3; *P*<.001; V=0.16) and 2023 (*χ*^2^_2_=409.5; *P*<.001; V=0.21). A significant but weak association was observed for the DB group in 2021 (*χ*^2^_1_=10.1; *P*=.001; φ=0.08) and a moderate effect size emerged in 2023 (*χ*^2^_3_=41.3; *P*<.001; V=0.14). The CX group showed a significant association in 2022 (*χ*^2^_2_=14.1; *P*<.001; V=0.10) and 2023 (*χ*^2^_2_=70.2; *P*<.001; V=0.10), though effect sizes remained weak throughout. BP had significant but weak associations between intervention and outcome for all 3 years. All significant chi-square test results are listed in Table 1, along with the analytic sample size. A complete report on all variables is available in Multimedia Appendix 4.

**Table 1:** Significant associations between covariates and outcome.

Measure and variable			Year		n		Chi-square (*df*)		*P* value		Effect size (φ or V)		Interpretation		
**Primary care**															
	Active PCP^a^ relationship	2021		5415		270.8 (1)		<.001		φ=0.22		Moderate			
	Active PCP relationship	2022		8445		14.1 (1)		<.001		φ=0.04		Weak			
	Active PCP relationship	2023		9544		265.7 (1)		<.001		φ=0.17		Weak			
	Ethnicity	2022		8445		41.4 (1)		<.001		φ=0.07		Weak			
	Ethnicity	2023		9544		46.8 (1)		<.001		φ=0.07		Weak			
	Gender	2021		5415		8.7 (1)		.003		φ=0.04		Weak			
	Gender	2022		8445		8.7 (1)		.003		φ=0.03		Weak			
	Gender	2023		9544		5.4 (1)		.02		φ=0.02		Weak			
	Intervention	2021		5415		1767.8 (3)		<.001		V=0.57		Large			
	Intervention	2022		8445		220.3 (2)		<.001		V=0.16		Moderate			
	Intervention	2023		9544		409.5 (2)		<.001		V=0.21		Moderate			
	Race group	2021		5415		8.8 (3)		.03		V=0.04		Weak			
	Race group	2022		8445		54.9 (3)		<.001		V=0.08		Weak			
	Race group	2023		9544		20.5 (3)		<.001		V=0.05		Weak			
	SVI^b^ quartile	2021		5415		25.2 (2)		<.001		V=0.07		Weak			
	SVI quartile	2022		8445		10.4 (2)		.006		V=0.04		Weak			
**D** **iabetes care**															
	Active PCP relationship	2021		1737		29.9 (1)		<.001		φ=0.13		Weak			
	Active PCP relationship	2022		1470		7.2 (1)		.007		φ=0.07		Weak			
	Gender	2022		1470		11.1 (1)		<.001		φ=0.09		Weak			
	Intervention	2021		1737		10.1 (1)		.001		φ=0.08		Weak			
	Intervention	2023		2216		41.3 (3)		<.001		V=0.14		Moderate			
	SVI quartile	2023		2216		18.6 (2)		<.001		V=0.09		Weak			
**Cancer screening**															
	Active PCP relationship	2021		4252		8.2 (1)		.004		φ=0.03		Weak			
	Active PCP relationship	2023		7571		4.4 (1)		.04		φ=0.02		Weak			
	Ethnicity	2022		3407		15.4 (1)		<.001		φ=0.07		Weak			
	Ethnicity	2023		7571		6.1 (1)		.01		φ=0.03		Weak			
	Gender	2021		4252		35.0 (1)		<.001		φ=0.09		Weak			
	Gender	2022		3407		5.1 (1)		.02		φ=0.04		Weak			
	Intervention	2022		3407		14.1 (2)		<.001		V=0.10		Weak			
	Intervention	2023		7571		70.2 (2)		<.001		V=0.10		Weak			
	Race group	2022		3407		22.7 (3)		<.001		V<0.01		Weak			
	SVI quartile	2023		7571		17.0 (2)		<.001		V=0.05		Weak			
**Blood pressure**															
	Active PCP relationship	2021		1484		6.8 (1)		.009		φ=0.07		Weak			
	Active PCP relationship	2022		1852		135.7 (1)		<.001		φ=0.27		Moderate			
	Gender	2022		1852		10.4 (1)		.001		φ=0.08		Weak			
	Intervention	2021		1484		17.0 (1)		<.001		φ=0.11		Weak			
	Intervention	2022		1852		41.0 (1)		<.001		φ=0.15		Weak			
	Intervention	2023		2542		58.1 (2)		<.001		V=0.03		Weak			
	SVI quartile	2021		1484		14.2 (2)		<.001		V=0.10		Weak			
	SVI quartile	2022		1852		21.7 (3)		<.001		V=0.11		Moderate			
	SVI quartile	2023		2542		34.3 (2)		<.001		V=0.12		Weak			

^a^PCP: primary care provider.

^b^SVI: social vulnerability index.

The PCP relationship was significantly associated with preventive care gap closure across several measure groups. In the PC group, a moderate association was observed in 2021 (*χ*^2^_1_=270.8; *P*<.001; φ=0.22), with significance maintained through 2023 (*P*<.001; φ=0.17). In the BP group, associations were significant in 2021 (*χ*^2^_1_=6.8; *P*=.009; φ=0.07) and stronger in 2022 (*χ*^2^_1_=135.7; *P*<.001; φ=0.27) but not sustained in 2023. The DB group followed a similar pattern, showing small but significant associations in 2021 (*χ*^2^_1_=29.9; *P*<.001; φ=0.13) and 2022 (*χ*^2^_1_=7.2; *P*=.007; φ=0.07), which was not sustained in 2023. The CX group showed inconsistent results, with weak associations in 2021 (*χ*^2^_1_=8.2; *P*=.004; φ=0.03) and 2023 (*χ*^2^_1_=4.4; *P*=.04; φ=0.02).

The association between SVI and compliance showed dynamic patterns across years. SVI demonstrated a consistent association with compliance in the BP group for all 3 years, peaking in 2022 with the strongest effect size (*χ*^2^_3_=21.7; *P*<.001; V=0.11). In contrast, the CX group (*χ*^2^_2_=17.0; *P*<.001; V=0.05) and the DB group (*χ*^2^_2_=18.6; *P*<.001; V=0.09) showed this association in 2023 only, both with small effect sizes. The same association weakened over time in the PC group, with significant, weak associations observed in 2021 (*χ*^2^_2_=25.2; *P*<.001; V=0.07) and 2022 (*χ*^2^_2_=10.4; *P*=.006; V=0.04), and no significant association detected in 2023.

Race was significantly associated with compliance in the PC group across all years, but effect sizes were negligible (Cramer V ranged from 0.04 to 0.08). A similar near-zero association was observed for the CX group (V=0.004) in 2022 only. Ethnicity demonstrated small but consistent associations with PC compliance in 2022 (*χ*^2^_1_=41.4; *P*<.001; φ=0.07) and 2023 (*χ*^2^_1_=46.8; *P*<.001; φ=0.07). In the CX group, ethnicity was associated in 2022 (*χ*^2^_1_=15.4; *P*<.001; φ=0.07) with a small effect, and in 2023 with a weaker effect (*χ*^2^_1_=6.1; *P*=.01; φ=0.03). No significant associations with race or ethnicity were observed in the DB or BP groups.

A significant but small and diminishing association between gender and compliance was noted in the PC group across all 3 years, with effect sizes ranging from 0.02 to 0.04. For the BP and DB groups in 2022 only, gender was significantly associated with compliance with weak effect sizes. Gender associations in the CX group are not reported because most measures in this group specifically targeted women.

### Regression Analysis of Compliance Predictors

**Figure 2:**
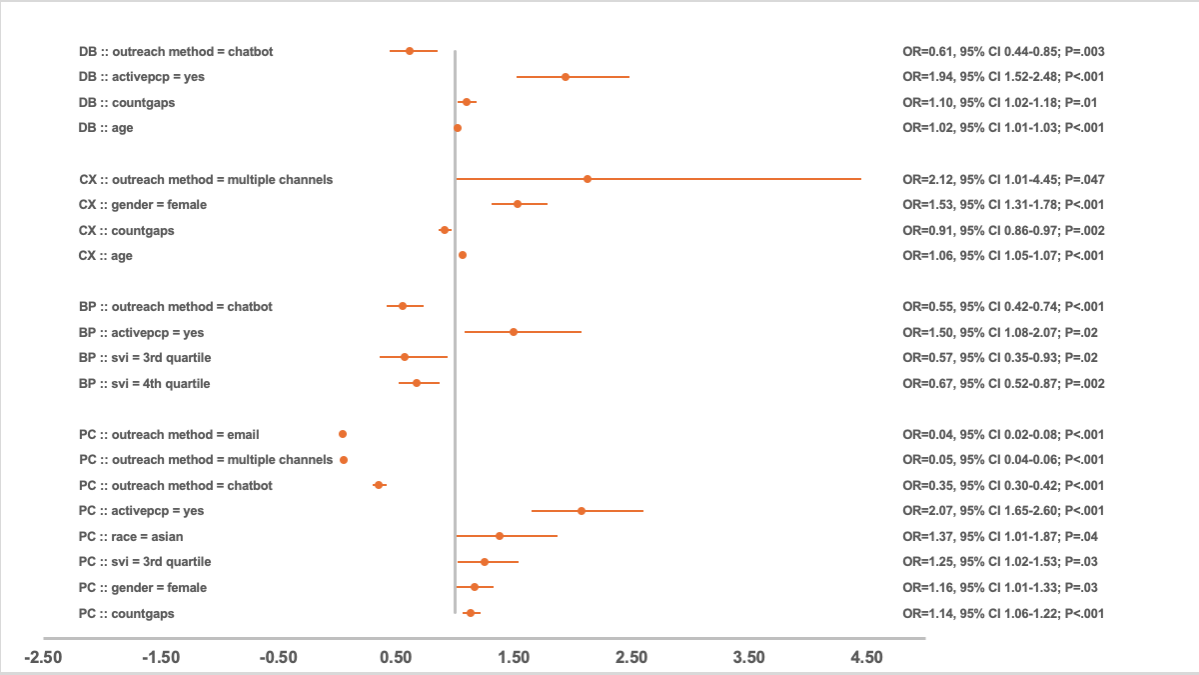
Odds ratio and 95% CI by significant predictors and measure group in 2021. BP: blood pressure; CX: cancer screening; DB: diabetes care; OR: odds ratio; PC: primary care; PCP: primary care provider; SVI: social vulnerability index.

**Figure 3:**
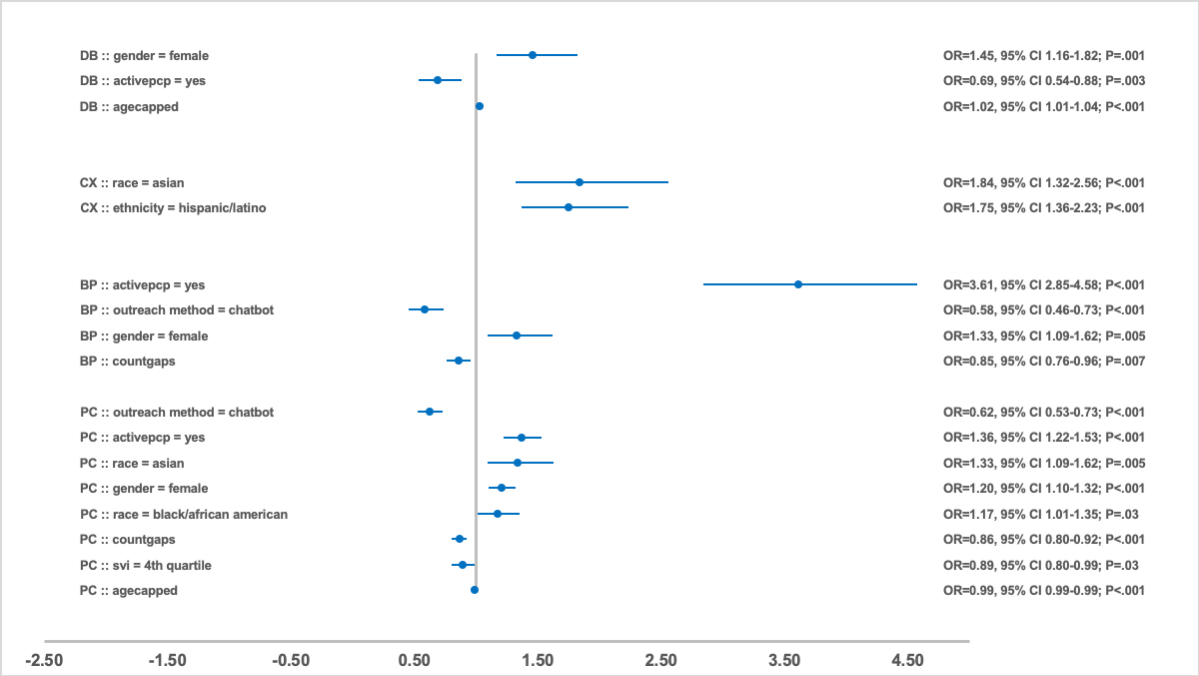
Odds ratio and 95% CI by significant predictors and measure group in 2022. BP: blood pressure; CX: cancer screening; DB: diabetes care; OR: odds ratio; PC: primary care; PCP: primary care provider; SVI: social vulnerability index.

**Figure 4:**
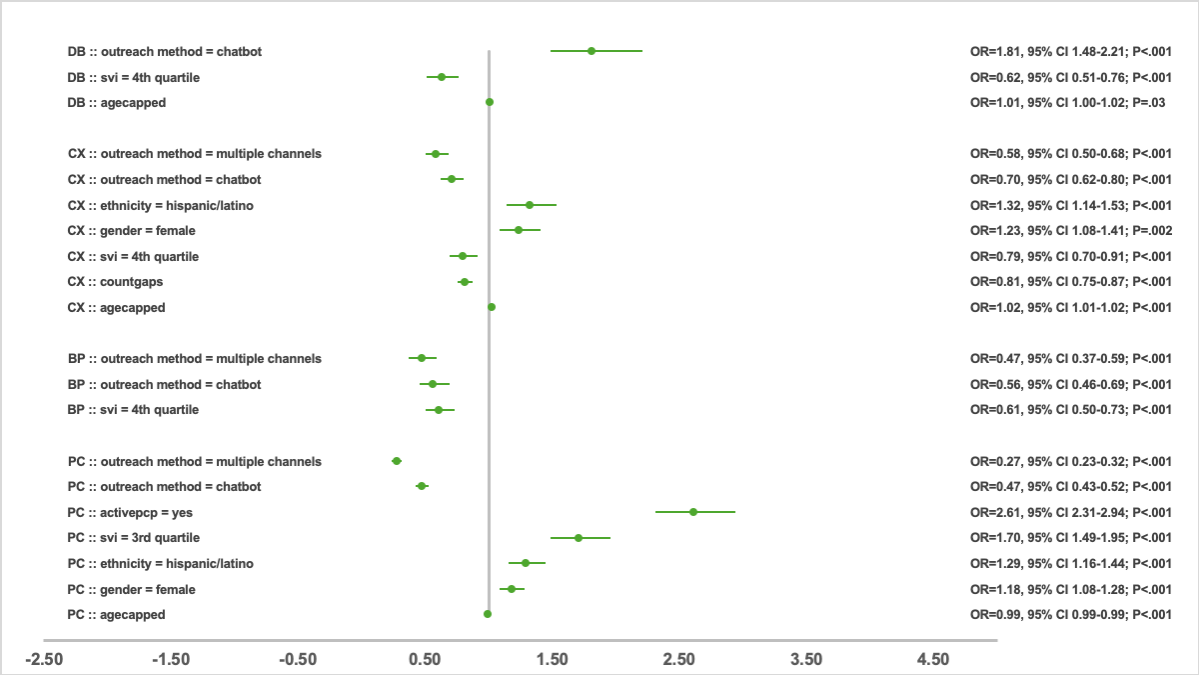
Odds ratio and 95% CI by significant predictors and measure group in 2023. BP: blood pressure; CX: cancer screening; DB: diabetes care; OR: odds ratio; PC: primary care; PCP: primary care provider; SVI: social vulnerability index.

After adjusting for other significant predictors, the outreach method remained significantly associated with compliance. Compared with phone call outreach, chatbot outreach consistently reduced the odds of gap closure across PC, BP, and CX measures, with a 30%-65% decrease in likelihood, as shown in Figures 2-4. An exception was observed in the DB group in 2023, where chatbot outreach outperformed phone calls (OR 1.81, 95% CI 1.48-2.21; *P*<.001). Multichannel campaigns were generally less effective than phone calls and chatbot outreach, except in the CX group in 2021, where multichannel outreach increased the odds of gap closure compared with phone calls (OR 2.12, 95% CI 1.01-4.44; *P*=.047). This was likely a reflection of the relatively smaller sample size that year, whereas subsequent years had greater representation of multichannel outreach, yielding narrower CIs.

Taking account of other covariates, having a PCP relationship was the strongest predictor (*P*<.001) for care gaps compliance in the PC measure group, with ORs 2.07 (95% CI 1.65-2.60), 1.36 (95% CI 1.22-1.53), and 2.61 (95% CI 2.31-2.94) for 2021, 2022, and 2023 respectively. BP and DB had similarly significant associations in 2021 (BP: OR 1.50, 95% CI 1.08-2.07; *P*=.02; DB: OR 1.94, 95% CI 1.52-2.48; *P*<.001) and 2022 (BP: OR 3.61, 95% CI 2.85-4.58; *P*<.001; DB: OR 0.69, 95% CI 0.54-0.88; *P*=.003), but this association was not sustained in 2023. The final model for the CX group did not surface the PCP relationship as a predictor.

The SVI quartile demonstrated a complex relationship with compliance in the PC group. Participants in the third quartile were more likely to close gaps in 2021 (OR 1.25, 95% CI 1.02-1.53; *P*=.03) and 2023 (OR 1.70, 95% CI 1.49-1.95; *P*<.001) compared with the less vulnerable first quartile. In contrast, in 2022, participants in the fourth quartile were less likely to close gaps (OR 0.89, 95% CI 0.80-0.99; *P*=.03) relative to the first quartile group. For the BP group, lower odds of gap closure were observed among the third quartile (OR 0.57, 95% CI 0.35-0.93; *P*=.02) and fourth quartile (OR 0.67, 95% CI 0.52-0.87; *P*=.002) in 2021, as well as the fourth quartile in 2023 (OR 0.61, 95% CI 0.50-0.73; *P*<.001). Similar patterns were noted for the CX and DB groups in 2023, where participants in the fourth quartile had reduced odds of closing gaps (CX: OR 0.79, 95% CI 0.70-0.91; *P*<.001; DB: OR 0.62, 95% CI 0.51-0.76; *P*<.001).

Women in the PC group had higher odds of compliance than men across all years, with ORs of 1.16 (95% CI 1.01-1.33; *P*=.03) in 2021, 1.20 (95% CI 1.10-1.32; *P*<.001) in 2022, and 1.18 (95% CI 1.08-1.28; *P*<.001) in 2023. Higher odds of gap closure for women were also observed in 2022 for the BP group (OR 1.33, 95% CI 1.09-1.62; *P*=.005) and DB group (OR 1.45, 95% CI 1.16-1.82; *P*=.001).

Asians were more likely to close gaps in PC group during 2021 (OR 1.37, 95% CI 1.01-1.87; *P*=.04) and 2022 (OR 1.33, 95% CI 1.09-1.62; *P*=.005) compared with White participants. They also showed higher odds of compliance in the CX group in 2022 (OR 1.84, 95% CI 1.32-2.56; *P*<.001). Race and ethnicity were not retained in the final model for BP and DB across all 3 years. However, ethnicity had a positive influence compliance in the CX group in 2022 (OR 1.75, 95% CI 1.36-2.23; *P*<.001) and 2023 (OR 1.32, 95% CI 1.14-1.53; *P*<.001), as well as in the PC group in 2023 (OR 1.29, 95% CI 1.16-1.44; *P*<.001).

Increasing age (capped at 89 years) showed a small, diminishing association with compliance. In the DB group, each additional year of age was associated with 1%-2% higher likelihood of gap closure, while in the CX group, the increased likelihood was 6% in 2021 and 2% in 2023. In contrast, in the PC group, older age was associated with a 1% lower likelihood of closing gaps in 2022 and 2023. Age was not a significant predictor in the final BP models across all 3 years. All percentage values for likelihood are reported based on the OR values in Figures 2-4.

An increase in the number of gaps was associated with a higher likelihood of gap closure in the DB group only in 2021 (OR 1.10, 95% CI 1.02-1.18; *P*=.01), with no significant effects observed in later years. Unlike the DB group, BP and CX groups showed an inverse relationship, where each additional gap decreased the likelihood of compliance (BP 2022: OR 0.85, 95% CI 0.76-0.96; *P*=.007; CX 2021: OR 0.91, 95% CI 0.86-0.97; *P*=.002; CX 2023: OR 0.81, 95% CI 0.75-0.87; *P*<.001). The PC group demonstrated inconsistency, with more gaps increasing the odds of compliance in 2021 (OR 1.14, 95% CI 1.06-1.22; *P*<.001) but decreasing them in 2022 (OR 0.86, 95% CI 0.80-0.92; *P*<.001). ORs, 95% CIs, and *P* values for all variables with significant association in the final regression models are presented in Figures 2-4 across the 3 years. Each point in these figures represents the OR for the listed cohort and variable group on the left, and the line through the point represents the CI. The actual numbers for OR, CI, and *P* value are listed on the right side. Reference values for each category include male, White, non-Hispanic/Latino, SVI first quartile, no active PCP, and phone outreach. Any subgroups excluded due to low volume (<10) for the year are not included in the regression analysis.

Model discrimination, as assessed by the area under the receiver operating characteristic curve (AUC), was highest for the PC measure group in 2021 (AUC=0.82), indicating good discriminatory ability. Other measure groups demonstrated modest discrimination, with AUCs ranging from 0.59 to 0.68 across years. In 2021, the model also achieved the highest classification accuracy for the PC group (78.6%), while the classification accuracy for other groups varied, ranging from 70.6% (BP) to 63.5% (CX) and 67% (DB).

Across all models, chi-square statistics were significant (*P*<.001), suggesting that the models were statistically associated with the outcomes. Nagelkerke *R*^2^ values, reflecting the proportion of variance explained, were highest in 2021 for the PC group (*R*^2^=0.41) and lower for other models, ranging from 0.02 to 0.13. Calibration, as assessed by the Hosmer-Lemeshow goodness-of-fit test, showed acceptable fit for some models (eg*,* CX in 2022 and 2023: *P*=.85 and *P*=.98; DB in 2022 and 2023: *P*=.34), but poorer fit in others (eg, PC in 2023: *P*<.001), indicating room for improvement in predicted versus observed outcomes alignment. Complete model diagnostics are provided in Table 2.

**Table 2:** Final regression model metrics for each measure group, 2021-2023.

Year and measure group		Chi-square (*df*)	*P* value	Nagelkerke *R*^2^	*P* value (Hosmer–Lemeshow)	Overall classification accuracy, %	AUC^a^
**2021**							
	PC^b^	1942.15 (9)	<.001	0.41	.02	78.6	0.82
	BP^c^	37.44 (5)	<.001	0.04	.10	70.6	0.59
	CX^d^	244.08 (6)	<.001	0.08	.002	63.5	0.65
	DB^e^	71.39 (4)	<.001	0.06	<.001	67.0	0.60
**2022**							
	PC	408.45 (10)	<.001	0.06	<.001	59.3	0.63
	BP	180.73 (4)	<.001	0.13	.21	67.7	0.68
	CX	48.79 (8)	<.001	0.02	.85	83.2	0.59
	DB	37.00 (4)	<.001	0.04	.34	69.3	0.60
**2023**							
	PC	882.59 (9)	<.001	0.12	<.001	60.7	0.67
	BP	90.76 (6)	<.001	0.05	.09	58.1	0.61
	CX	145.14 (7)	<.001	0.03	.98	78.9	0.60
	DB	68.64 (6)	<.001	0.04	.34	55.6	0.60

^a^AUC: area under the receiver operating characteristic curve.

^b^PC: primary care.

^c^BP: blood pressure.

^d^CX: cancer screening.

^e^DB: diabetes care.

## Discussion

### Key Findings

Outreach modality remains the key modifiable lever for influencing preventive care gap closure strategies. However, its impact is declining in the PC group across the 3 years, is marginal in the BP and CX group, and needs continued studies in the DB group to establish a trend and sustained evidence of association. In VBC, where outreach for gaps in care is conducted at scale, even smaller effect sizes are worth noting, as they highlight the need for ongoing research to further understand nuances between measure groups and outreach technology preferences for optimizing operational efficiencies.

Among the outreach methods, phone calls demonstrated a stronger positive impact on outcomes compared to chatbots. This may be attributed to the chatbot’s deterministic, rule-based design, which lacks the interactivity and adaptability of more advanced artificial intelligence (AI)–powered conversational agents. These results are also in agreement with the recent findings from McDowell et al [20], who presented health communication preferences from their study to show that while the majority of participants preferred SMS text messaging communication in interactions with family and friends, their preference in communicating with health care providers was primarily by use of the phone call. Even though the chatbot design for this study was a web-based chat conversation, it was initiated through an SMS text message seeking consent for further communication. As noted by Fanaroff [21], SMS text messaging–based communication, though universally accessible, may face barriers in health communications due to privacy laws such as the HIPAA (Health Insurance Portability and Accountability Act)–related security constraints imposed during implementation, which can create barriers like requiring multiple clicks to get to the core of the message. This can erode patient trust in digital health technologies, especially as incidences of SMS text message phishing attacks have increased in the last 5 years [22]. The 2023 diabetes group exception may point to a self-management culture within DB, suggesting potential for enhanced impact through digital engagement. It certainly points to a need for segmentation by measure groups when designing outreach strategies, as well as a need for further exploration of the underlying factors driving these emerging behavioral patterns.

Sustained PCP relationships more than doubled the odds of closing gaps in PC, reinforcing the understanding that it facilitates preventive measures and use in agreement with previous findings [23]. However, the effect reversed for diabetes in 2022, suggesting disease-specific pathways (eg, endocrinology follow-up may supplant the PCP role for glycemic monitoring). The lack of association between PCP relationships and BP, DB, and CX in the 2023 regression models suggests that outreach strategies should be customized by measure and specifically address the distinct impact of PCP relationships within each measure group.

Socioeconomic vulnerability appears to be an emerging barrier. The strengthening association and effect size for this covariate in the BP group over time suggest that an equity-focused resource allocation for hypertension is warranted. The association that emerged in the CX and DB groups in 2023 cannot be ignored. These results are consistent with evidence that shows neighborhood-level disadvantage suppresses screening adherence, with Montgomery et al [24] presenting one recent example of such evidence in a cancer cohort. However, the differential effect between third and fourth quartile participants in the PC group contradicts the usual linear-deprivation narrative. This highlights the need to study community-level resilience factors and changing attitudes toward PC since the 2020 pandemic.

Other demographic predictors had a relatively smaller impact. Race and ethnicity became significant variables for PC and CX outcomes in 2022 and 2023 with small effect sizes. Specifically, the positive direction of the OR for minority groups like Asians and Hispanics is surprising and suggests the need for further targeted research into culture-based practices that promote positive outcomes, so outreach strategies can be culturally tailored for better effectiveness. Gender effects in the PC group are consistently small but show higher odds of compliance among women, agreeing with prior findings [11]. In contrast, gender effects in the BP and DB groups are inconsistent across the 3 years, while interpretation within the CX group is limited due to 2 of its 3 measures being restricted to female participants.

The overall compliance rate difference between the various measure groups does indicate some weight for the burden of completion. For example, the BP measure can be completed with a single PCP visit during the year, whereas CX measures require multiple engagements with health care, which may include imaging appointments or laboratory work in addition to follow-up visits. Complex measures should be complemented with additional support or touchpoints for optimal outreach strategies.

### Limitations

This study has several limitations that should be considered when interpreting the findings. First, although the analysis draws from a large sample of individuals with preventive care gaps, the retrospective observational design limits the explanatory power of the findings. Additionally, the outreach method distribution was not uniform across measure groups; for instance, email-only and multichannel outreach cohorts did not consistently meet inclusion thresholds, restricting comparative analyses within certain subgroups. Because outreach assignments followed operational workflows rather than randomization, some selection bias may exist. Exclusions for incomplete or low-volume subgroups were applied to ensure valid statistical testing and consistent data quality. While these steps may modestly limit generalizability, the retained sample remained representative of the health system’s attributed population, supporting the internal validity of findings. Generalizability is therefore most applicable to similar real-world VBC settings.

An additional limitation of this study was the presence of unmeasured variables that may account for differences in the efficiency of outreach methods. Factors such as participants’ language preference, digital literacy, barriers to completion, comorbidities, and other psychosocial or contextual influences were not consistently captured across all outreach methods, likely contributing to the limited variance explained by several models. Although classification accuracy was acceptable in some cases (eg, 78.6% for PC in 2021), lower performance metrics, including the area under the receiver operating characteristic curve (eg, 0.59 for CX in 2022), indicate potential model under specification. Future research should incorporate more granular clinical, behavioral, and sociodemographic variables to enhance understanding and optimization of outreach effectiveness.

### Recommendations for Practice

Even though this is not a randomized trial, the findings from this study apply to a large representative population, therefore several practical recommendations can be made to enhance the design and implementation of outreach strategies for value-based cohorts. Phone calls consistently outperformed chatbots and multichannel campaigns in driving preventive care gap closure across most measure groups and years. Health systems should continue to prioritize phone-based outreach, particularly for high-impact measure groups such as PC, while evaluating digital methods as supplementary rather than primary channels until they are more effective or better personalized. The study highlights meaningful variations in outreach effectiveness by measure group and socioeconomic status. To maximize effectiveness, outreach strategies should be customized according to the specific clinical area and complexity of the care gap, such as differentiating approaches for PC, chronic disease management, and more complex screening measures. For example, the stronger performance of chatbot outreach in the DB group in 2023 suggests that measure-specific digital readiness may guide outreach channel selection.

We also recommend enhancing chatbots for better engagement. Chatbots are an emerging technology that offers better scalability than phone calls. The rule-based design of the chatbot used in this study likely limited its effectiveness. Transitioning to more advanced, AI-powered conversational agents that support interactive dialogue and user personalization could improve patient engagement and outcomes [25].

Socioeconomic vulnerability was a significant and increasing barrier to care gap closure, particularly in the BP group. Health systems should adopt equity-focused outreach strategies that prioritize high-SVI communities for additional support, such as community health worker involvement or tailored messaging. This is especially important in measure groups with heavier burdens of completion, like CX or chronic disease monitoring.

The relatively low explanatory power of the models underscores the need to expand the range of included variables. Incorporating clinical data (eg, comorbidities and medication adherence), behavioral factors (eg, health beliefs and prior screening history), and social determinants of health (eg, transportation access and housing stability) could significantly enhance the precision of predictive models used to guide outreach prioritization.

### Conclusion

In conclusion, this study found that there is variability in the effectiveness of chatbots in outperforming traditional outreach methods for preventive screenings. The findings confirm that outreach modality continues to be a key, modifiable factor in closing preventive care gaps, with phone-based outreach consistently outperforming chatbots and multichannel strategies across most measure groups. However, the effectiveness of each outreach method varied notably by clinical context and patient subgroup, highlighting the need for a segmented outreach design that accounts for differences in patient preferences, health conditions, and burden of gap completion. The limited effectiveness of deterministic, rule-based chatbots points to the potential value of transitioning to more adaptive, AI-enabled engagement tools, particularly those capable of personalized communication and responsiveness to digital literacy. Socioeconomic vulnerability remained a key barrier, with differing trends between the third and fourth SVI quartiles indicating that vulnerability is not a linear predictor of behavior. Incorporating structured data on preferences, barriers, and comorbidities will improve model accuracy. Future research using prospective, randomized designs should further evaluate outreach modality, language, and communication preferences to guide inclusive, high-impact preventive care.

## Appendix

Multimedia Appendix 1 Measure group volume of participants and gaps per year of study. Each cell lists participant volume with measure gaps volume in brackets (n).

Multimedia Appendix 2 Table S1: Distribution table for final analysis of PC group 2021-2023. Table S2: Distribution table for final analysis of BP group 2021-2023. Table S3: Distribution table for final analysis of CX group 2021-2023. Table S4: Distribution table for final analysis of DB group 2021-2023.

Multimedia Appendix 3 Compliance rate by measure group 2021-2023.

Multimedia Appendix 4 Table A: All Associations between covariates and outcome.

Multimedia Appendix 5 Eligibility criteria for each included measure for 2021-2023.

Multimedia Appendix 6 Distribution (%) of gaps by outreach method and measure group 2021-2023.

## Abbreviations

AI: artificial intelligence
AUC: area under the receiver operating characteristic curve
BP: blood pressure
CMS: Centers for Medicare & Medicaid Services
CX: cancer screening
DB: diabetes care
EMR: electronic medical record
HEDIS: Healthcare Effectiveness Data and Information Set
HIPAA: Health Insurance Portability and Accountability Act
OR: odds ratio
PC: primary care
PCP: primary care provider
SVI: social vulnerability index
VBC: value-based care

## Data Availability

The datasets analyzed in this study were derived from health system operational data and accessed under applicable data use agreements that restrict public dissemination, even in deidentified form. Due to such contractual, ethical, and governance constraints, the data are not publicly available. Access may be considered upon reasonable request to the corresponding author and is subject to approval by the data-owning institution and execution of an appropriate data use agreement.
