# Supplementary figures and images for: Chatbot Outreach in Value-Based Preventive Care: Retrospective Analysis
Source: JMIR Med Inform. 2026 Feb 12;14:e81370. doi: 10.2196/81370 (PMC12946782; doi:10.2196/81370)

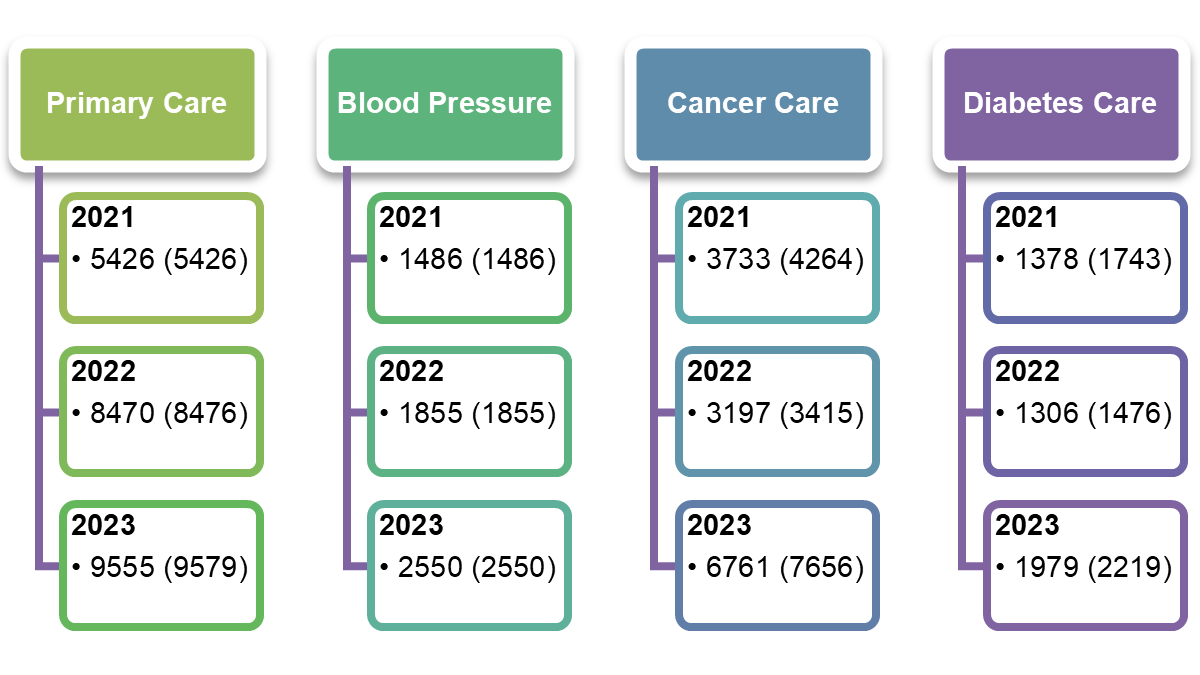

Supplement: Multimedia Appendix 1 [file medinform_v14i1e81370_app1.png]

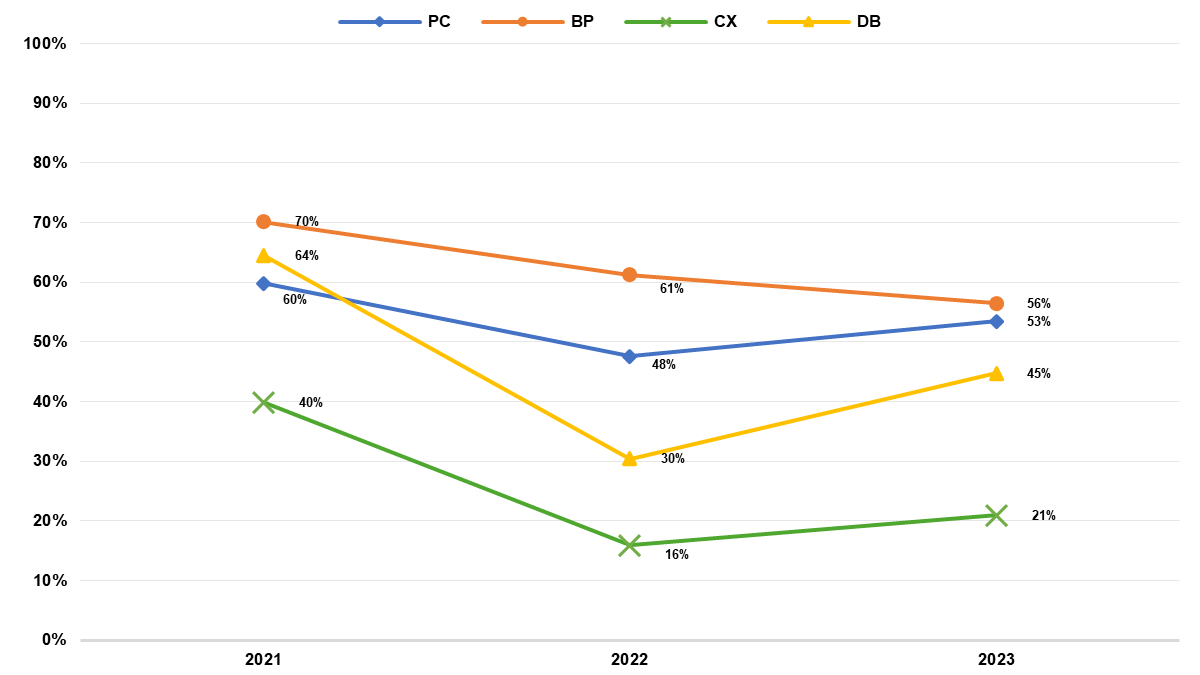

Supplement: Multimedia Appendix 3 [file medinform_v14i1e81370_app3.png]

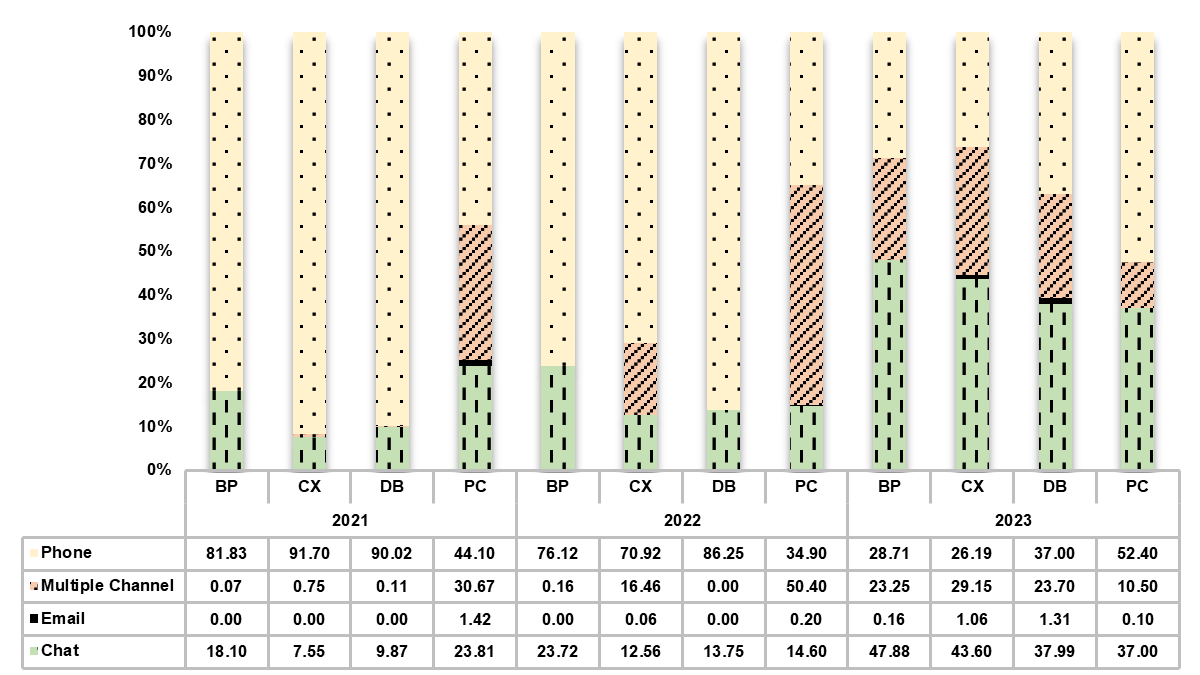

Supplement: Multimedia Appendix 6 [file medinform_v14i1e81370_app6.png]
