# Supplementary material for: Chatbot Outreach in Value-Based Preventive Care: Retrospective Analysis
Source: JMIR Med Inform. 2026 Feb 12;14:e81370. doi: 10.2196/81370 (PMC12946782; doi:10.2196/81370)
Supplement: Multimedia Appendix 5 [file medinform_v14i1e81370_app5.docx]

# Multimedia Appendix 5: Eligibility criteria for each included measure for all 3 years of study.

| Measure name | Measure requirement | Measure timeframe | Eligible population | Does not meet criteria |
| --- | --- | --- | --- | --- |
| Annual Wellness Visit | Documented annual wellness visit with physical examination | Current calendar year | Any age | Visit addressing only acute or chronic conditions |
| Pediatric Well-Care Visit | Annual Well Child Visit (CPE) | Current calendar year | Children aged 3–21 years | Child younger than 3 years at time of visit |
| Blood Pressure Screening | Most recent recorded blood pressure value <140/90 with documented date^a^ | Current calendar year | Adults aged 18–75 years | Patient-reported values or missing date |
| Breast Cancer Screening | Qualifying breast cancer screening evidence^b^ | Current or prior calendar year | Women aged 50–74 years | Imaging without full date |
| Cervical Cancer Screening | Qualifying cervical cancer screening evidence^c^ | Variable by age and test interval | Women aged 21–65 years | Test without valid timeframe or date |
| Colorectal Cancer Screening (2021–2022) | Approved colorectal screening test with valid interval^d^ | Variable by test interval^d^ | Adults aged 50–75 years | Test without documented date |
| Colorectal Cancer Screening (2023) | Approved colorectal screening test with valid date documentation^d^ | Variable by test interval^d^ | Adults aged 45–75 years | Test without documented date |
| Diabetes Care: HbA1c Poor Control (>9.0%) | Most recent HbA1c result >9% with full date^e^ | Current calendar year | Adults aged 18–75 years | Point-of-care glucose testing or patient reported values |
| Diabetes Care: HbA1c Control (<8.0%) | Most recent HbA1c result <8% with full date^e^ | Current calendar year | Adults aged 18–75 years | Point-of-care glucose testing or patient reported values |
| Diabetes Care: HbA1c Testing | Any documented HbA1c test with full date^e^ | Current calendar year | Adults aged 18–75 years | Patient-reported test only |
| Diabetes Care: Retinal Eye Exam | Documented dilated retinal examination with date and ophthalmologist name^f^ | Current or prior calendar year | Adults aged 18–75 years | Optometrist visit only |
| Diabetes Care: Nephropathy Assessment | Documented urine protein testing^g^ | Current calendar year | Adults aged 18–75 years | Patient-reported testing |
| Kidney Health Evaluation for Patients With Diabetes | Documented eGFR test and urine albumin-creatinine ratio^h^ | Current calendar year | Adults aged 18–85 years | Missing either required test |

1. Blood pressure measurement may be obtained during a nurse visit if documented in the medical record.
2. Includes mammography with full date or documentation of bilateral mastectomy at any time including patient reported documentation with MM/YY is sufficient.
3. Includes Pap smear alone for women aged 21–64 years, Pap smear with HPV testing for women aged 30–64 years or documented complete hysterectomy. Patient reported dates with MM/YY are sufficient.
4. Approved tests include fecal immunochemical test (1 year), FIT-DNA (3 years), sigmoidoscopy (5 years), colonoscopy (10 years), or complete colectomy (any year).
5. HbA1c must be a laboratory result with full date; point-of-care glucose testing or patient reported value does not qualify.
6. Includes dilated eye examination by an ophthalmologist or a negative retinopathy exam in the prior year also qualifies.
7. Includes urine microalbumin, albumin, or protein testing with full date.
8. Both eGFR and urine albumin-creatinine ratio must be documented in the same measurement year.
